# The Physiology of the Teeth Properly Applied to Their Care and Preservation

**Published:** 1852-01

**Authors:** 


*The Physiology of the Teeth Properly applied to their Care and Preservation.* By Joseph Snake. Second Edition. Loughman & Co., page 76.

Mr. Snake has long been favorably known in England, as the uncompromising enemy to dental quackery, he has been repeatedly engaged in giving popular lectures upon the science of the art, and the looseness of character of many of those who pretend to be professionals, therefore, in presenting a second edition of his little work to the public, he has done his brethren and the public good service—the subjects are all scientifically and judiciously treated, particularily that when giving advice upon the insertion of artificial teeth, he observes, "the difficulties to be overcome, differ almost in every case; but whatever character they may assume, the above shows how necessary it is that the mouth should be rendered perfectly healthy, before an artificial restoration can be either permanently comfortable or beneficial.

